# A Two-Component DNA-Prime/Protein-Boost Vaccination Strategy for Eliciting Long-Term, Protective T Cell Immunity against *Trypanosoma cruzi*

**DOI:** 10.1371/journal.ppat.1004828

**Published:** 2015-05-07

**Authors:** Shivali Gupta, Nisha J. Garg

**Affiliations:** 1 Department of Microbiology and Immunology, School of Medicine, University of Texas Medical Branch (UTMB), Galveston, Texas, United States of America; 2 Department of Pathology, School of Medicine, University of Texas Medical Branch (UTMB), Galveston, Texas, United States of America; 3 Institute for Human Infections and Immunity and the Sealy Center for Vaccine Development, University of Texas Medical Branch (UTMB), Galveston, Texas, United States of America; University of Iowa, UNITED STATES

## Abstract

In this study, we evaluated the long-term efficacy of a two-component subunit vaccine against *Trypanosoma cruzi* infection. C57BL/6 mice were immunized with TcG2/TcG4 vaccine delivered by a DNA-prime/Protein-boost (D/P) approach and challenged with *T*. *cruzi* at 120 or 180 days post-vaccination (dpv). We examined whether vaccine-primed T cell immunity was capable of rapid expansion and intercepting the infecting *T*. *cruzi*. Our data showed that D/P vaccine elicited CD4^+^ (30-38%) and CD8^+^ (22-42%) T cells maintained an effector phenotype up to 180 dpv, and were capable of responding to antigenic stimulus or challenge infection by a rapid expansion (CD8>CD4) with type 1 cytokine (IFNγ^+^ and TFNα^+^) production and cytolytic T lymphocyte (CTL) activity. Subsequently, challenge infection at 120 or 180 dpv, resulted in 2-3-fold lower parasite burden in vaccinated mice than was noted in unvaccinated/infected mice. Co-delivery of IL-12- and GMCSF-encoding expression plasmids provided no significant benefits in enhancing the anti-parasite efficacy of the vaccine-induced T cell immunity. Booster immunization (bi) with recombinant TcG2/TcG4 proteins 3-months after primary vaccine enhanced the protective efficacy, evidenced by an enhanced expansion (1.2-2.8-fold increase) of parasite-specific, type 1 CD4^+^ and CD8^+^ T cells and a potent CTL response capable of providing significantly improved (3-4.5-fold) control of infecting *T*. *cruzi*. Further, CD8^+^T cells in vaccinated/bi mice were predominantly of central memory phenotype, and capable of responding to challenge infection 4-6-months post bi by a rapid expansion to a poly-functional effector phenotype, and providing a 1.5-2.3-fold reduction in tissue parasite replication. We conclude that the TcG2/TcG4 D/P vaccine provided long-term anti-*T*. *cruzi* T cell immunity, and bi would be an effective strategy to maintain or enhance the vaccine-induced protective immunity against *T*. *cruzi* infection and Chagas disease.

## Author Summary

Chagas disease, caused by *Trypanosoma cruzi* infection, represents the third greatest tropical disease burden in the world. No vaccine or suitable treatment is available for control of this infection. Based upon several studies we have conducted, we believe that TcG2 and TcG4 candidate antigens that are highly conserved in *T*. *cruzi*, expressed in clinically relevant forms of the parasite, and recognized by both B and T cell responses in multiple hosts, are an excellent choice for subunit vaccine development. In this study, we demonstrate that the delivery of TcG2 and TcG4 as a DNA-prime/protein-boost vaccine provided long-term protection from challenge infection, and this protection was associated with elicitation of long-lived CD8^+^ effector T cells. The longevity and efficacy of vaccine could be enhanced by booster immunization. We believe that this is the first report demonstrating a) a subunit vaccine can be useful in achieving long-term protection against *T*. *cruzi* infection and Chagas disease, and b) the effector T cells can be long-lived and play a role in vaccine elicited protection from parasitic infection.

## Introduction

Chagas disease is prevalent in almost all Latin American countries, including Mexico and Central America [1]. Currently, ~11–18 million individuals are infected worldwide, and ~13,000 children and adults die annually because of the clinical complications of *T*. *cruzi*-induced heart disease and lack of effective treatments [2]. The vectorial, autochthonous, and congenital transmission of *T*. *cruzi* exists in the United States, where >300,000 infected individuals can potentially transfer infection through blood or organ donation [3–5]. When considered from a global perspective, Chagas disease represents the third greatest tropical disease burden after malaria and schistosomiasis [6].

Before setting the goal of vaccine development against any disease, an important question is whether vaccination is an economically viable approach with desirable health benefits. With regard to *T*. *cruzi* infection, the research community has pushed for a vaccine that can achieve complete parasite elimination from the host. However, several studies, including our published reports (reviewed in [7]), testing the efficacy of subunit vaccines have resulted in findings that vaccine-induced immunity can provide a reduction in tissue parasite burden associated with variable degrees of control of acute or chronic disease symptoms. The vaccine mediated control of infection and disease in experimental studies generally resembled that noted in 60–70% of the chagasic patients that remained seropositive and maintained residual parasites for their entire lives, but did not develop a clinically symptomatic form of the disease [2]. Further, recent computer simulation modeling of the impact of a prophylactic vaccine for Chagas disease showed that a vaccine would provide net cost savings (along with health benefits), even when the risk of infection is only 1%, vaccine efficacy is only 25%, and the cost of a vaccine is US$20 or lower [8]. Thus, it is ethically appropriate to consider a satisfactory vaccination goal to reduce the frequency and severity of clinical disease by decreasing the extent of persistent parasite burden; and accordingly, continuing efforts towards developing a vaccine against *T*. *cruzi* infection and Chagas disease are economically justifiable.

We have employed a computational/bioinformatics approach for unbiased screening of the *T*. *cruzi* genome database and identification of 11 potential candidates [9,10]. Through rigorous analysis over a period of several years, we determined that three candidates (TcG1, TcG2, TcG4) were maximally relevant for vaccine development [11]. These candidates were highly conserved in clinically relevant *T*. *cruzi* strains, expressed (mRNA/protein) in infective trypomastigote and intracellular amastigote stages of *T*. *cruzi*, and recognized by immunoglobulins and CD8^+^T cells in multiple *T*. *cruzi*-infected hosts [10,11].

We have examined the protective efficacy of TcG1, TcG2 and TcG4 (individually or in combination) in mice. Our data showed that co-delivery of the three antigens elicited additive immunity and protection from *T*. *cruzi* infection than was noted with individual candidate antigens [11]. Delivery of the 3-component vaccine by a DNA-prime/DNA-boost approach was less effective than the heterologous DNA-prime/protein-boost (D/P) approach in eliciting protective immunity [11–13]. Mice challenged with *T*. *cruzi* immediately after immunization with the 3-component D/P vaccine were capable of controlling 90–97% of the acute parasitemia and tissue parasite burden, and, subsequently, inflammatory infiltrate and tissue fibrosis were particularly absent in the heart and skeletal muscle of vaccinated mice [13].

In this study, we have sought to determine the long-term efficacy of the subunit vaccine against *T*. *cruzi* infection. We included TcG2 and TcG4 in the vaccine, as these antigens were most potent in eliciting parasite-specific antibody and CD8^+^T cell immunity [11–13]. Mice were immunized with TcG2/TcG4 vaccine delivered by the D/P approach, and we examined whether a) the 2-component D/P vaccine primed T_H_1 CD4^+^T cells and generated a stable pool of CD8^+^T memory cells, and b) the vaccine-primed T cells were capable of rapid expansion and intercepting the infecting *T*. *cruzi*. We also determined if c) the 2-component D/P vaccine primed immunity is enhanced by co-delivery of IL-12 and GM-CSF cytokine adjuvants, or d) a booster immunization (bi) several months after the second vaccine dose was effective in providing better protection from *T*. *cruzi* infection.

## Results

### Two-component DNA-prime/protein-boost (D/P) vaccine elicited long-lived anti-*T*. *cruzi* T cell immunity

**Fig 1:**
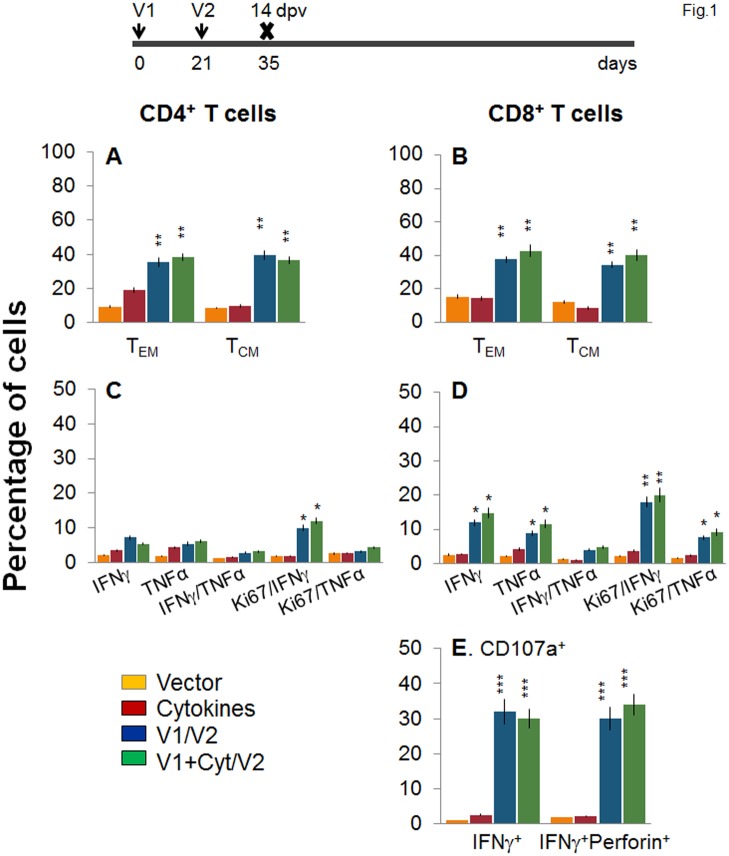
Two-component, DNA-prime/protein-boost (D/P) vaccine elicits poly-functional T cell response in mice. C57BL/6 mice were immunized with an empty vector, cytokines only, or D/P vaccine (V1 dose: TcG2- and TcG4-encoding plasmids ± IL-12- and GMCSF-expression plasmids; and V2 dose: recombinant TcG2 and TcG4 proteins), as detailed in Materials and Methods. **(A&B)** Splenocytes were obtained at 14 days post-vaccination (dpv) and *ex vivo* labeled with PE-conjugated anti-CD4, FITC-conjugated anti-CD8, PerCPCy5.5-conjugated anti-CD62L, and APC-conjugated anti-CD44 antibodies. Shown are the PE^+^CD4^+^ ***(A)*** and FITC^+^CD8^+^ ***(B)*** T cell subsets that were of effector/effector memory (T_EM:_ CD44^+^CD62L^-^) and central memory (T_CM,_ CD44^+^CD62L^+^) phenotype, determined by flow cytometry. **(C-E)** Splenocytes were *in vitro* stimulated for 48 h with TcG2 and TcG4 recombinant antigens and labeled with fluorescent-conjugated antibodies. The mean percentage of PE^+^CD4^+^ ***(C)*** and FITC^+^CD8^+^ ***(D)*** T cells that were IFN*γ* ^+^ (e-Fluor) and/or TNFα^+^ (Cy5) with or without Ki67^+^ (PerCPCy 5.5) phenotype are shown. **(E)** The percentage of antigen-specific CD8^+^IFNγ^+^ T cells that were CD107a^+^ (Alexa-Fluor 488) and/or perforin^+^ (APC) was acquired by flow cytometry analysis. In all figures, data are presented as mean ± SD (n = 8/group, triplicate observations per experiment). Significance is presented as *p<0.05, **p<0.01, ***p<0.001 (vaccinated versus non-vaccinated or vaccinated/infected versus non-vaccinated/infected).

D/P vaccination resulted in 60–70% expansion of splenic cell number, observed at day 14 and 120 post-vaccination (S1 Table). To examine the T cell profile primed by the TcG2- and TcG4-encoding D/P vaccine, splenocytes from immunized mice were submitted to flow cytometry analysis before and after *in vitro* stimulation with recombinant antigens. Splenocytes were gated for CD4^+^ and CD8^+^ T cells and analyzed for surface markers of effector/effector memory (T_EM_, CD44^+^CD62L^-^) and central memory (T_CM_, CD44^+^CD62L^+^) phenotype. The immediate early T cell response to D/P vaccine, measured at 14 days post vaccination (dpv), was evidenced by a significant increase in *ex vivo* levels of CD4^+^ (36–39% of total, 3.8–4.7-fold) and CD8^+^ (34–43% of total, 2.5–3.3-fold) T_EM_ and T_CM_ cells, respectively, in D/P vaccinated mice when compared to that noted in non-vaccinated controls (Fig 1A and 1B, p<0.01). The vaccine-primed CD4^+^ and CD8^+^ T cells exhibited antigen-specific activation and proliferation (Ki67^+^), and were positive for IFN*γ* (10–12% and 18–20%, respectively) and TNFα (3.2–4.4% and 7.6–9.2%, respectively) cytokines (Fig 1C and 1D, p<0.05–0.01). Transport of CD107a/b integral membrane proteins to the plasma membrane of effector T cells is required for the cytolytic activity mediated by perforin and granzyme and the release of IFN*γ* which exerts pleiotropic effects to suppress intracellular pathogens. Flow cytometry studies showed a high frequency of the vaccine-primed CD8^+^T cells were CD107a^+^IFN*γ* ^+^perforin^+^ (30–34%, Fig 1E, p<0.001).

**Fig 2:**
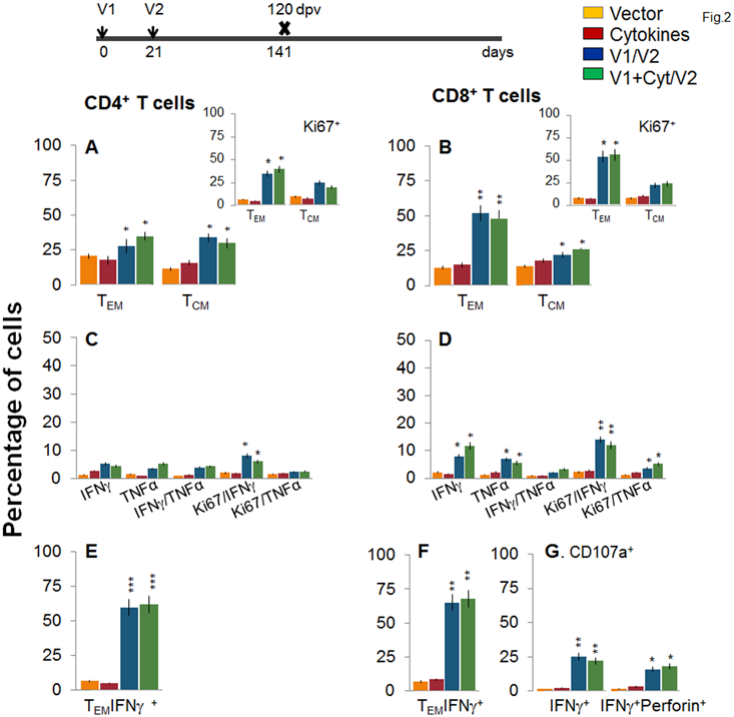
Stability and effector phenotype of D/P vaccine elicited T cells in mice. Mice were vaccinated as in Fig 1 and harvested at 120 days after the 2^nd^ vaccine dose. **(A&B)** Splenocytes were *ex-vivo* labeled with fluorescence-conjugated antibodies and analyzed for CD4^+^ ***(A)*** and CD8^+^ ***(B)*** T cell subsets (T_EM:_ CD44^+^CD62L^-^; T_CM:_ CD44^+^CD62L^+^) by flow cytometry. Insets show D/P vaccine-primed T cell subsets that proliferated (Ki67^+^) in response to antigenic stimulus. **(C-G)** Splenocytes were *in vitro* stimulated for 48 h in the presence of TcG2 and TcG4 recombinant antigens, and incubated with fluorescent-conjugated antibodies as described in Fig 1. Shown are bar graphs of antigen-specific CD4^+^ ***(C&E)*** and CD8^+^ ***(D&F)*** T cell subsets that were proliferative (Ki67^+^, ***C&D***) with T effector ***(E&F)*** phenotype and produced IFN*γ* and/or TNFα cytokines. The percentage is shown of antigen-specific CD8^+^CD107a^+^ T cells that were IFN*γ* ^+^perforin^-^ or IFN*γ* ^+^perforin^+^ ***(G)***.

Next, we examined the stability and effector phenotype of T cells in vaccinated mice at 120 dpv. The *ex vivo* frequency of CD4^+^ T_EM_ and T_CM_ (28–35%) was slightly decreased, while that of CD8^+^ T_EM_ cells (48–52%) was increased, with a simultaneous decline in CD8^+^ T_CM_ cells (22–26%) in vaccinated mice harvested at 120 dpv (compare Fig 2A and 2B with Fig 1A and 1B). The vaccine-elicited long-lived T cells proliferated upon antigenic stimulation (Ki67^+^, insets in Fig 2A and 2B), though the frequency of cytokine-producing CD4^+^T cells (Ki67^+^IFN*γ* ^+^: 6–8% versus 10–12%; Ki67^+^TNFα^+^: 2.2–2.4% versus 3–4%) and CD8^+^T cells (Ki67^+^IFN*γ* ^+^: 12–14% versus 18–20%; Ki67^+^TNFα^+^: 3.6–5.2% versus 8–9%) at 120 dpv, as compared to that noted at 14 dpv, was significantly decreased (compare Fig 2C and 2D with Fig 1C and 1D). Yet, 62–68% of the IFN*γ* producing CD4^+^ and CD8^+^ T cells were CD44^+^CD62L (T_EM_ phenotype, Fig 2E and 2F, p<0.01–0.001), and 25% and 16–18% of the CD8^+^IFN*γ* ^+^ T cells were CD107a^+^ and CD107a^+^perforin^+^, respectively, indicating their cytolytic phenotype (Fig 1G, p<0.05–0.01). Together, the data presented in Figs 1 and 2 suggested that the 2-component D/P vaccine primed CD4^+^ and CD8^+^ T_EM_/T_CM_ cells were long-lived, and responded to antigenic stimulus with type 1 cytokine production and CTL activity. Co-delivery of cytokine adjuvants did not change the frequency or phenotype of the vaccine-primed T cells in immunized mice. No antigen-specific expansion of CD4^+^ and CD8^+^ T cells was observed in control mice given empty vector or cytokine adjuvants only.

**Fig 3:**
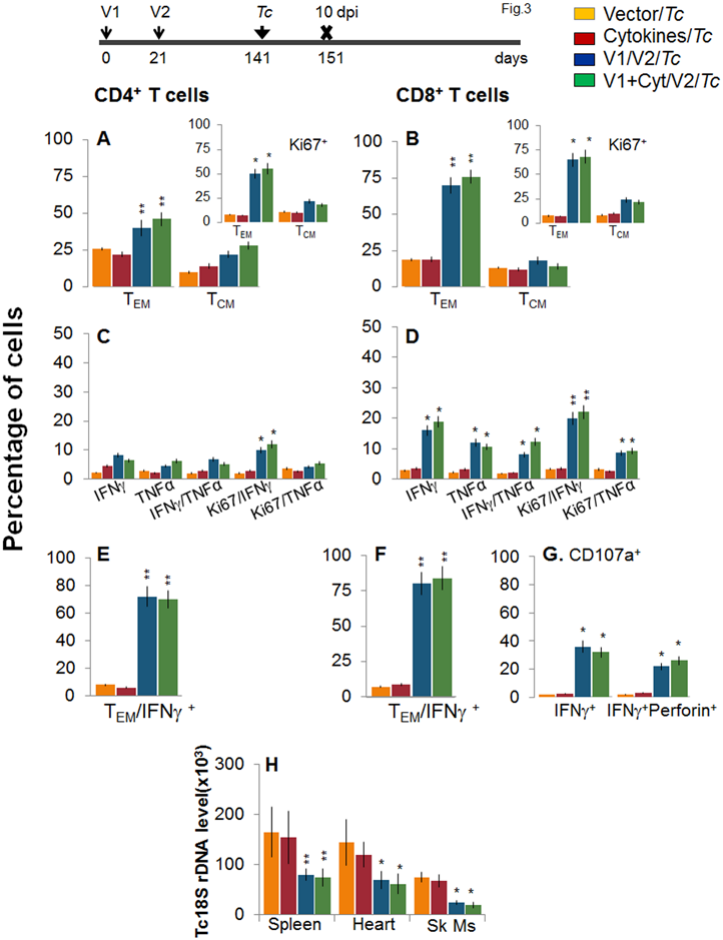
Recall of the D/P vaccine elicited, long-lived T cells after challenge infection with *T*. *cruzi*. Mice were vaccinated as in Fig 2, and at 120 days post-vaccination, challenged with *T*. *cruzi* trypomastigotes (10,000 parasites/mouse), as described in Materials and Methods. Mice were harvested at 10 days post-infection (dpi). **(A&B)** Bar graphs show *ex vivo* percentage of PE^+^CD4^+^ ***(A)*** and FITC^+^CD8^+^ ***(B)*** splenic T cell subsets (T_EM:_ CD44^+^CD62L^-^; T_CM:_ CD44^+^CD62L^+^) in vaccinated/infected mice. Insets show D/P vaccine-primed T cell subsets that proliferated (Ki67^+^) in response to *T*. *cruzi* infection (controls: non-vaccinated/infected mice). **(C-F)** Splenic cells from vaccinated/infected and non-vaccinated/infected mice were *in vitro* stimulated for 48 h in the presence of TcG2 and TcG4 recombinant antigens, and then labeled with fluorescent-conjugated antibodies, as above. Shown are the mean percentage of CD4^+^ ***(C&E)*** and CD8^+^ ***(D&F)*** T cells that responded to *T*. *cruzi* infection with production of IFN*γ* and/or TNFα and were proliferative (Ki67^+^, ***C&D***) with T effector ***(E&F)*** phenotype. **(G)** The percentage of *T*. *cruzi*-specific CD8^+^IFN*γ* ^+^T cells that were CD107a^+^perforin^-^ or CD107a^+^perforin^+^ are shown (± D/P vaccine). **(H) Tissue parasite burden**. Mice were challenged at 120 days post D/P vaccination and harvested at 10 dpi. Total DNA was isolated from spleen, heart and skeletal muscle (Sk Ms) tissue sections and submitted to real-time PCR amplification of *Tc*18SrDNA sequence (normalized to murine *GAPDH*).

To determine if the vaccine-elicited, long-lived T cells were responsive to *T*. *cruzi*, mice were challenged at 120 dpv and harvested at 10 days pi. Irrespective of vaccination status, all mice exhibited a significant (15-20-fold) expansion of splenic cell number, the maximum expansion being observed in vaccinated/infected mice (S1 Table). *Ex vivo* flow cytometry analysis of splenocytes showed the expansion of proliferating (Ki67^+^) CD4^+^ and CD8^+^ T_EM_ cells by 1.3–1.6-fold in response to *T*. *cruzi* infection in vaccinated mice (Fig 3A and 3B and insets, compare with Fig 2A and 2B, p<0.05–0.01). Further, vaccinated mice exhibited up to 57%, 29% and 79% increase in antigen-specific IFN*γ* ^+^, TNFα^+^, and IFN*γ* ^+^TNFα^+^ CD4^+^T cells, respectively, following challenge infection (compare Fig 3C with 2C); and a majority (70–72%) of the IFN*γ* ^+^CD4^+^T cells were proliferative (Ki67^+^) with effector (CD44^+^CD62L^-^) phenotype (Fig 3C and 3E, p<0.05–0.01). An expansion of vaccine-induced CD8^+^T cells in response to challenge infection was evidenced by a >2-fold increase in IFN*γ* ^+^ and TNFα^+^ and a 4-fold increase in IFN*γ* ^+^TNFα^+^ CD8^+^T cells (compare Fig 3D with Fig 2D, p<0.05–0.01); of which 80–84% exhibited proliferative (Ki67^+^) and effector (CD44^+^CD62L^-^) phenotype (Fig 3D and 3F, p<0.01). Likewise, vaccinated mice, in response to challenge infection, exhibited a 40–91% expansion of antigen-specific CD8^+^IFN*γ* ^+^T cells that were also CD107a^+^ or CD107a^+^perforin^+^ (compare Fig 3G with Fig 2G, p<0.05). When compared to non-vaccinated/infected mice, vaccinated/infected mice exhibited an 8- to 10-fold higher frequency of antigen-specific IFN*γ* ^+^ effector (CD44^+^CD62L^-^) CD4^+^ and CD8^+^ T cells and CD8^+^ cytolytic T cells (Fig 3E–3G, p<0.001). Quantitative PCR showed, respectively, 2.2-fold and 2-3-fold decline in peripheral (S1A Fig) and tissue (spleen, heart, skeletal muscle; Fig 3H, p<0.05–0.01) levels of *T*. *cruzi* in vaccinated/infected mice when compared to that noted in non-vaccinated/infected controls. Together, these data suggested that the D/P vaccine-elicited, long-lived T cells rapidly expanded (CD8>CD4) with a type 1 effector phenotype in response to challenge infection, and were capable of controlling *T*. *cruzi* infection.

### Booster immunization (bi) enhanced the recall T cell immunity against *T*. *cruzi*

**Fig 4:**
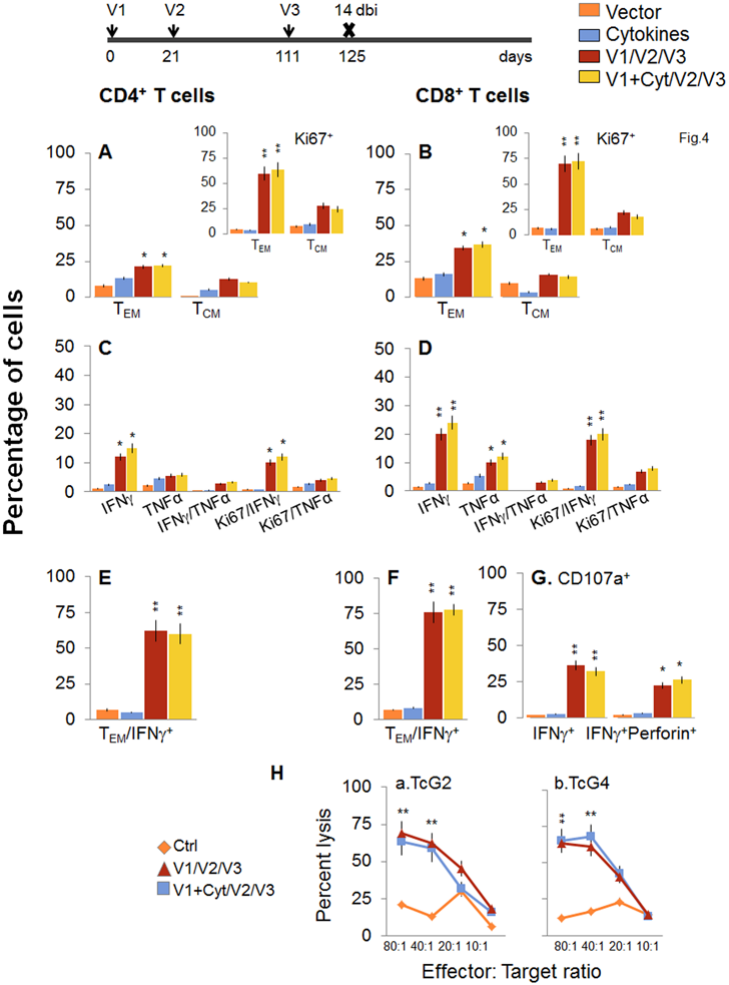
D/P vaccine elicited, long-lived T cells exhibited enhanced antigen-specific, poly-functional phenotype after booster immunization (bi). C57BL/6 mice were immunized at day 0 with V1 (TcG2- and TcG4-encoding plasmids ± IL-12- and GMCSF-expression plasmids), at day 21 with V2 (recombinant TcG2 and TcG4 proteins), and then at day 111 with V3 (booster dose, recombinant TcG2 and TcG4 proteins). Mice were harvested at 14 days after the booster immunization (dbi). Splenocytes were stimulated in the presence or absence of TcG2 and TcG4 recombinant antigens, labeled with fluorescent-conjugated antibodies, and analyzed by flow cytometry. **(A&B)** *Ex vivo* percentage of CD4^+^ ***(A)*** and CD8^+^ ***(B)*** splenic T cell subsets (T_EM:_ CD44^+^CD62L^-^; T_CM:_ CD44^+^CD62L^+^) in vaccinated/bi mice (Insets: antigen-specific Ki67^+^ T cell subsets in vaccinated/bi mice). **(C-G)** Bar graphs of antigen-specific CD4^+^ ***(C&E)*** and CD8^+^ ***(D&F)*** T cell subsets with their intracellular cytokine (IFN*γ* ^+^, TNFα^+^) profile that were proliferative (Ki67^+^, ***C&D***) with T effector ***(E&F)*** phenotype. The percentage of antigen-specific CD8^+^CD107a^+^ T cells that were IFN*γ* ^+^perforin^-^ and IFN*γ* ^+^Perforin^+^ ***(G)*** in vaccinated/bi mice are shown. **(H) Cytolytic activity**. Splenocytes were *in vitro* stimulated with recombinant antigens and used as effectors for their ability to lyse EL4 target cells exposed to GFP^+^ recombinant MVA encoding TcG2 *(H*.*a)* or TcG4 *(H*.*b)* antigens (controls: target cells incubated with empty MVA).

Next, we determined if a bi would enhance the vaccine elicited T cell immunity and provide better control of *T*. *cruzi* infection. For this, mice were immunized with the 2-component D/P vaccine as above, and given a booster dose of TcG2/TcG4 recombinant proteins before challenge infection. The booster-immunized mice exhibited 2-3-fold higher number of splenic cells as compared to non-vaccinated mice (S1 Table). When analyzing splenic T cell frequency, we observed an overall decline in the splenic frequency of CD4^+^ (T_EM_: 21.3–22.2% versus 28–35%, T_CM_: 10.5–12.8% versus 30–34%) and CD8^+^ (T_EM_: 34.5–36.8% versus 48–52%, T_CM_: 14.4–16% versus 22–26%) T cells at 14 days post bi, when compared to that noted before bi (compare Fig 4A and 4B with Fig 2A and 2B). However, splenocytes from vaccinated/bi mice *in vitro* stimulated with recombinant TcG2/TcG4 proteins exhibited 1.6–3.4-fold and 1.4–3.2-fold higher frequencies of the cytokine (IFN*γ* and/or TNFα) producing CD4^+^ and CD8^+^ T cells, respectively, as compared to that noted in vaccinated (but not bi) mice (compare Fig 4C and 4D with Fig 2C and 2D, all p<0.05–0.01). Approximately, 60–83% of the antigen-specific IFN*γ*- or TNFα-producing CD4^+^T cells and 80–90% of the IFN*γ*-producing CD8^+^T cells exhibited a proliferative (Ki67^+^), effector (CD44^+^CD62L^-^) phenotype (Fig 4E and 4F, p<0.01). Importantly the frequency of CD8^+^CD107a^+^ and CD8^+^CD107a^+^perforin^+^ cells secreting IFN*γ* was increased by 37.5–45% post bi (compare Fig 4G with Fig 2G, p<0.05–0.01).

To validate the antigen-specific cytotoxic activity of the CD8^+^T lymphocytes in vaccinated/bi mice, spleen cells *in vitro* stimulated with recombinant antigens were used as effectors and tested for their ability to lyse EL4 cells exposed to GFP^+^ rMVA encoding TcG2 or TcG4 antigens or empty MVA. A significant level of antigen-specific cytolytic activity (range 56–69%, effector-to-target cell ratio, 80: 1 or 40:1) was evident in vaccinated/bi mice (Fig 4H, p<0.01. Splenocytes from mice immunized with vector only or cytokines only did not show CTL activity against any of the antigen-sensitized target cells (Fig 4H).

**Fig 5:**
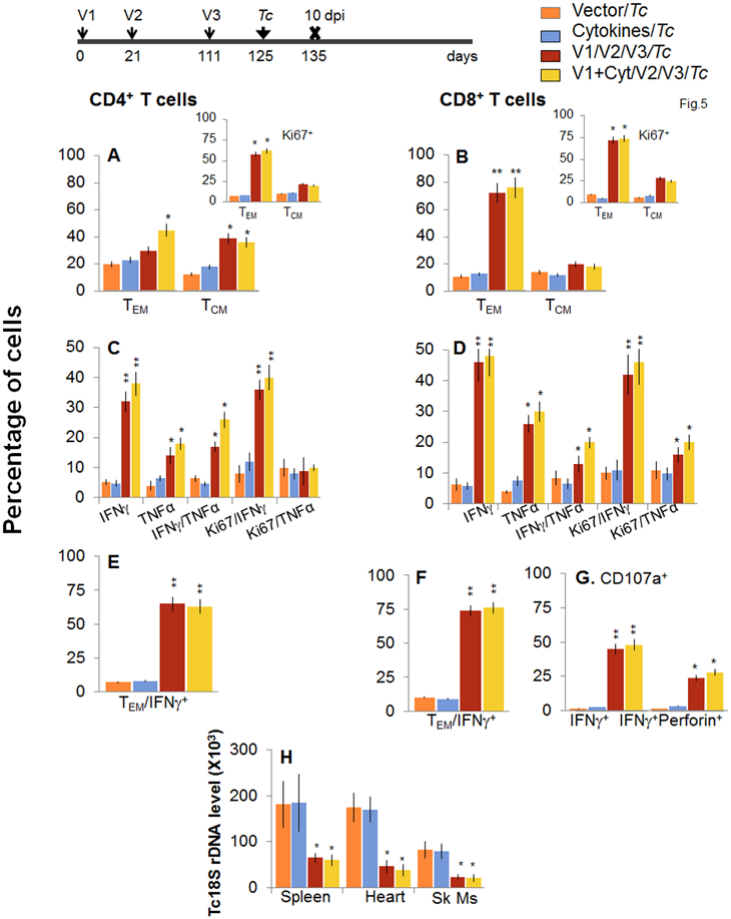
Booster immunization (bi) expanded the parasite-specific recall T cell response that provided superior protection from *T*. *cruzi* infection. Mice were immunized with D/P vaccine followed by a booster dose as in Fig 4, and then, 14 days later, challenged with *T*. *cruzi*. Mice were harvested at 10 days pi. Splenocytes were *in vitro* stimulated in the presence or absence of TcG2 and TcG4 recombinant antigens, labeled with fluorescence-conjugated antibodies and analyzed by flow cytometry. **(A&B)** *T*. *cruzi*-specific CD4^+^ ***(A)*** and CD8^+^ ***(B)*** splenic T cell subsets in vaccinated/bi mice at 10 days pi. Insets show antigen-specific T cell subsets that proliferated (Ki67^+^) in response to *T*. *cruzi* infection in vaccinated/bi mice. **(C-F)** Shown are the mean percentage of CD4^+^ ***(C&E)*** and CD8^+^ ***(D&F)*** T cells that responded to *T*. *cruzi* infection with production of IFN*γ* and/or TNFα cytokines and were proliferative (Ki67^+^, ***C&D***) with T effector ***(E&F)*** phenotype in vaccinated/bi mice. **(G)** Percentage of *T*. *cruzi*-specific CD8^+^CD107a^+^ that were IFN*γ* ^+^perforin^-^ or IFN*γ* ^+^perforin^+^ in booster-immunized/infected mice. **(H) D/P vaccine/bi was highly effective in controlling tissue parasite burden**. Total DNA was isolated from spleen, heart and skeletal muscle (Sk Ms) tissue sections of vaccinated/bi/infected mice, and real time PCR amplification of *Tc18SrDNA* sequence was performed. Bar graphs show the *Tc18SrDNA* level normalized to murine *GAPDH*.

When mice were challenged with *T*. *cruzi* immediately after bi, a potent expansion of parasite-specific T cells was evidenced by a 1.4-2-fold increase in *ex vivo* frequency of CD4^+^ and CD8^+^ T_EM_ cell populations (compare Fig 5A and 5B with Fig 4A and 4B, p<0.05–0.01). Further, splenocytes of vaccinated/bi mice, in response to challenge infection, exhibited a strong increase in *T*. *cruzi*-specific IFN*γ* ^+^ or TNFα^+^ (2.0–3.1-fold) and IFN*γ* ^+^TNFα^+^ (4.3–7.8-fold) CD4^+^ and CD8^+^ T cells, that were also Ki67^+^ with effector (CD44^+^CD62L^-^) phenotype (compare Fig 5C–5F with Fig 4C–4F, p<0.05–0.01). The D/P-vaccinated/bi mice exhibited a larger expansion of total splenic cells (S1 Table) and T cells than was noted in the D/P-vaccinated mice in response to challenge infection, as was evidenced by a significantly higher (1.6–2.8-fold, p<0.05–0.01) frequency of proliferating and non-proliferating IFN*γ* ^+^, TNFα^+^, and IFN*γ* ^+^TNFα^+^ CD4^+^ and CD8^+^ T cells (compare Fig 5 with Fig 3). Subsequently, vaccinated/bi mice exhibited a 2.5-fold decline in peripheral (S1B Fig) and 3–3.8-fold decline in tissue (spleen, heart and skeletal muscle) levels of *Tc18SrDNA* in comparison to that noted in non-vaccinated/infected mice (Fig 5H, p<0.05–0.01). Together, the data presented in Figs 4 and 5, along with that presented in Figs 2 and 3, suggested that bi was effective in expanding the 2-component D/P vaccine-elicited type 1 cytokine producing T cells and CD8^+^ CTLs that provided better protection from challenge infection than was observed with 2-component D/P vaccine only.

### Longevity and functional capability of vaccine/bi elicited T cells in providing protection from *T*. *cruzi* infection

**Fig 6:**
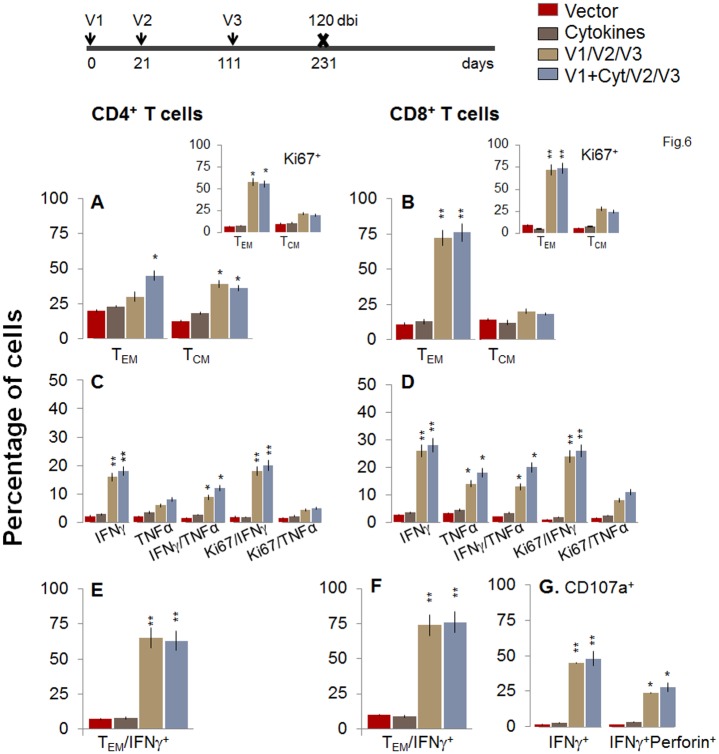
Two-component D/P vaccine/bi elicited T cells retain the antigen-specific functional profile at 120 days post bi. Mice were immunized with D/P vaccine followed by a booster dose as in Fig 4, and then harvested at 120 days after booster dose. Splenocytes were stimulated in the presence or absence of TcG2 and TcG4 recombinant antigens, labeled with fluorescent-conjugated antibodies, and analyzed by flow cytometry. **(A&B)** *Ex vivo* percentage of CD4^+^ ***(A)*** and CD8^+^ ***(B)*** splenic T cell subsets (T_EM:_ CD44^+^CD62L^-^; T_CM:_ CD44^+^CD62L^+^) at 120 days post bi (Insets: antigen-specific Ki67^+^ T cell subsets). **(C-G)** Bar graphs of CD4^+^ ***(C&E)*** and CD8^+^ ***(D&F)*** T cell subsets that proliferated (Ki67^+^, ***C&D***) or exhibited T_EM_ ***(E&F)*** phenotype with production of intracellular cytokines (IFN*γ*, TNFα) in vaccinated mice at 120 days post bi. Shown in ***(G)*** are the percentages of antigen-specific CD8^+^CD107a^+^T cells that were IFN*γ* ^+^perforin^-^ and IFN*γ* ^+^perforin^+^ ***(G)*** in D/P-vaccinated mice at 120 days post bi.

**Fig 7:**
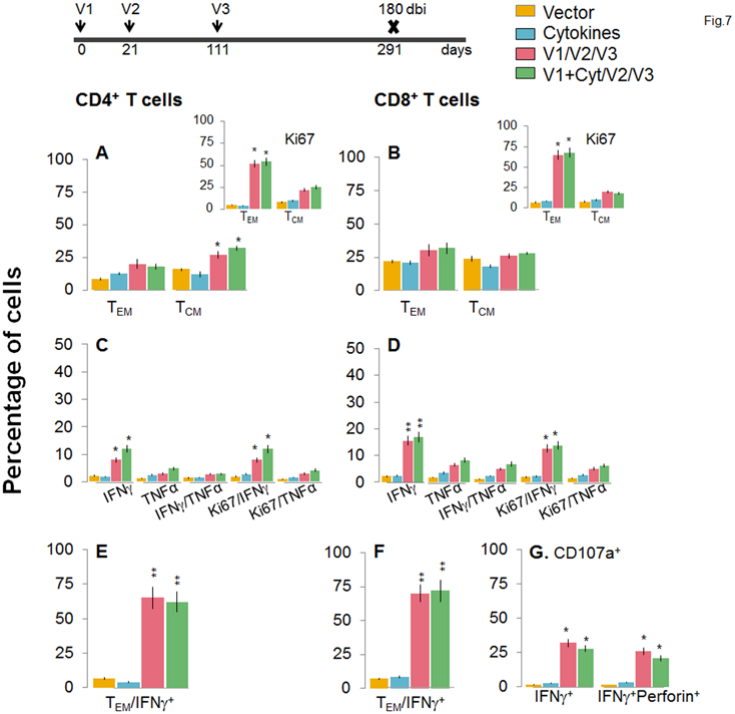
A decline in the frequency of D/P vaccine/bi elicited, long-lived CD4^+^ and CD8^+^ T cells occurred by 180 days post bi. Mice were immunized with D/P vaccine followed by a booster dose as in Fig 4, and then harvested 180 days later. Splenocytes were stimulated in the presence or absence of TcG2 and TcG4 recombinant antigens, labeled with fluorescent-conjugated antibodies, and analyzed by flow cytometry. **(A&B)** *Ex vivo* percentage of CD4^+^ ***(A)*** and CD8^+^ ***(B)*** splenic T cell subsets (T_EM:_ CD44^+^CD62L^-^; T_CM:_ CD44^+^CD62L^+^) at 180 day post bi (Insets: antigenic-specific Ki67^+^ T cell subsets). **(C-G)** Bar graphs of CD4^+^ ***(C&E)*** and CD8^+^ ***(D&F)*** T cell subsets that proliferated (Ki67^+^, ***C&D***) or exhibited T_EM_ ***(E&F)*** phenotype with production of intracellular cytokines (IFN*γ* and/or TNFα) in vaccinated mice at 180 days post bi. Shown in ***(G)*** are the percentages of antigen-specific CD8^+^CD107a^+^ T cells that were IFN*γ* ^+^perforin^-^ and IFNα^+^perforin^+^ ***(G)*** in vaccinated mice at 180 days post bi.

To evaluate the stability and effector phenotype of the T cells elicited by the 2-component D/P vaccine and bi, mice were harvested at 120 or 180 days post bi. As above, the booster-immunized mice continued to maintain 2-3-fold higher number of splenic cells at 120 or 180 days post bi as compared to non-vaccinated mice (S1 Table). The *ex vivo* frequency of CD4^+^ T_CM_ cells was increased by 3.1–3.4-fold and 2.1–3.2-fold at 120 and 180 days post-bi, respectively, while that of CD4^+^ T_EM_ cells was increased modestly (1.4-2-fold) at 120 days post bi only, when compared to that noted in mice harvested at 14 days post bi (compare Figs 6A and 7A with Fig 4A). *Ex vivo* CD8^+^T cells of T_EM_ phenotype at 120 days post bi (72–76%, Fig 6B) contracted by 2.4-fold at 180 days post bi (Fig 7B). In contrast, CD8^+^T cells of T_CM_ phenotype were increased by 1.4-fold at 120–180 days post bi compared to that noted in mice harvested at 14 days post bi (compare Figs 6B and 7B with Fig 4B).

To determine if long-lived vaccine/bi elicited T cells are functional, splenocytes from vaccinated/bi mice were harvested at 120 or 180 days post bi, *in vitro* stimulated with recombinant antigens, and analyzed by flow cytometry. We noted a modest decline in the frequency of antigen-specific Ki67^+^CD4^+^T cells at 120 days (56–58%) and 180 days (52–54%) post bi when compared to that noted at 14 days (60–64%) post bi (compare Figs 6A and 7A with Fig 4A). The splenic cells from vaccinated mice harvested at 120 days post bi exhibited a ~2-4-fold larger expansion of proliferative (Ki67^+^) and non-proliferative (Ki67^-^) IFN*γ* ^+^ and IFN*γ* ^+^TNFα^+^ CD4^+^T cells in response to *in vitro* antigenic stimulus than was observed with splenic cells from mice harvested at 14 or 180 days post bi (compare Fig 6C with Figs 7C and 4C, p<0.05–0.01). The CD8^+^T cells, at 120 days post bi, exhibited a similar level of antigen-specific proliferative (Ki67^+^) capacity, but a significantly higher capacity for type 1 cytokine (IFN*γ* and/or TNFα) production and lytic activity (IFN*γ* ^+^CD107a^+^perforin^-^ or IFN*γ* ^+^CD107a^+^perforin^+^) than was noted at 14 days post bi (compare Fig 6D, 6F, and 6G with Fig 4D, 4F, and 4G, p<0.05–0.01). At 180 days post bi, the percentage of CD8^+^T cells capable of proliferating and producing type 1 cytokines with lytic activity contracted by at least 30% in comparison to that noted at 120 days post bi (compare Fig 7D, 7F, and 7G with Fig 6D, 6F, and 6G). Together, the data presented in Figs 6 and 7 (along with Fig 4) suggested that the D/P vaccine/bi elicited CD4^+^ and CD8^+^ T cells were stable, the frequency of T_CM_ cells was enhanced at 120–180 days post bi; and these CD4^+^ and CD8^+^ T cells were capable of rapidly responding to antigenic stimulus with proliferation and activation of type 1 cytokine production and cytolytic profile.

**Fig 8:**
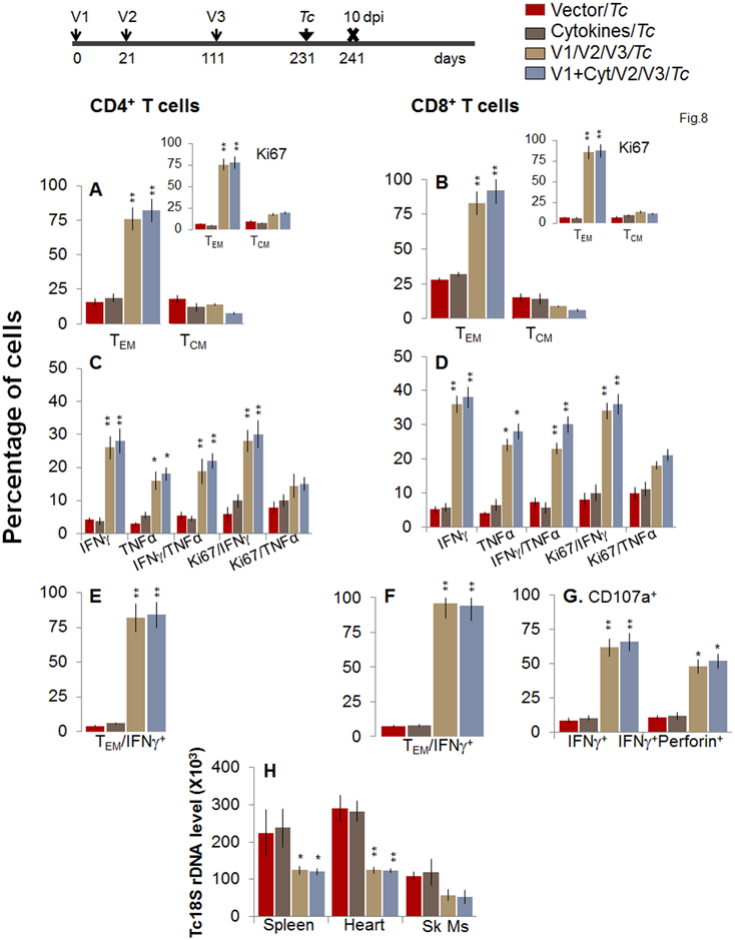
Booster-immunized mice expand the recall T cell response capable of controlling *T*. *cruzi* infection at 120 days post bi. Mice were immunized with D/P vaccine followed by a booster dose as in Fig 4, and then, at 120 days post bi, infected with *T*. *cruzi*. Mice were harvested 10 days after the challenge infection, and splenocytes were stimulated in the presence or absence of TcG2 and TcG4 recombinant antigens, labeled with fluorescent-conjugated antibodies, and analyzed by flow cytometry. **(A&B)** *T*. *cruzi*-specific CD4^+^ ***(A)*** and CD8^+^ ***(B)*** splenic T cell subsets in vaccinated/bi mice infected at 120 days post bi. Insets show *T*. *cruzi*-specific, proliferating (Ki67^+^) T cell subsets in vaccinated mice challenged at 120 days post bi. **(C-F)** Shown are the mean percentage of CD4^+^ ***(C&E)*** and CD8^+^ ***(D&F)*** T cells that responded to *T*. *cruzi* infection by producing IFN*γ* and/or TNFα cytokines and were proliferative (Ki67^+^, ***C&D***) with a T effector ***(E&F)*** phenotype in mice challenged at 120 days post bi. **(G)** The percentage of *T*. *cruzi*-specific CD8^+^CD107a^+^ that were IFN*γ* ^+^perforin−^-^ or IFN*γ* ^+^perforin^+^ in mice that were infected at 120 days post bi. **(H) Tissue parasite burden**. Bar graphs of *Tc18SrDNA* levels, normalized to murine *GAPDH*, were developed by using a real time quantitative PCR approach.

Finally, we determined whether long-lived T cells present in vaccinated/bi mice were capable of responding to *T*. *cruzi* infection. For this, at 120 or 180 days post bi, mice were challenged with *T*. *cruzi* and splenic cell characterization performed at day 10 pi. When challenged at 120 days post bi, mice exhibited a similar expansion of CD4^+^T_EM_ cells capable of producing IFN*γ* and/or TNFα cytokines as was noted in mice challenged at 14 days post bi (compare Fig 8A, 8C, and 8E with Fig 5A, 5C and 5E, p<0.05–0.01). The CD8^+^T cells were maintained at a very high frequency (72–76%) in vaccinated mice at 120 days post bi (Fig 6B), and expanded only by 15–21% post challenge infection (Fig 8B). Importantly, in mice challenged at 120 days post bi, 36–38%, 24–28%, and 23–30% of the antigen-specific CD8^+^T cells were IFN*γ* ^+^, TNFα^+^ or IFN*γ* ^+^TNFα^+^, respectively, and 48–52% of the IFN*γ* ^+^CD8^+^T cells were also CD107^+^perforin^+^ (Fig 8D, 8F, and 8G, p<0.05–0.01). These results suggested a significant expansion of cytokine producing (35–77%) CD8^+^T cells with a cytolytic phenotype (2-fold increase) in mice challenged at 120 days post bi in comparison to that noted in mice challenged at 14 days post bi (compare Fig 8D, 8F, and 8G with Fig 5D, 5F, and 5G, p<0.05–0.01). Subsequently, vaccinated/bi mice challenged 120 days post bi exhibited a significant control (1.5–2.3-fold) of peripheral (S1B Fig) and tissue (Fig 8H, p<0.05–0.01) parasites in comparison to the non-vaccinated/infected mice.

**Fig 9:**
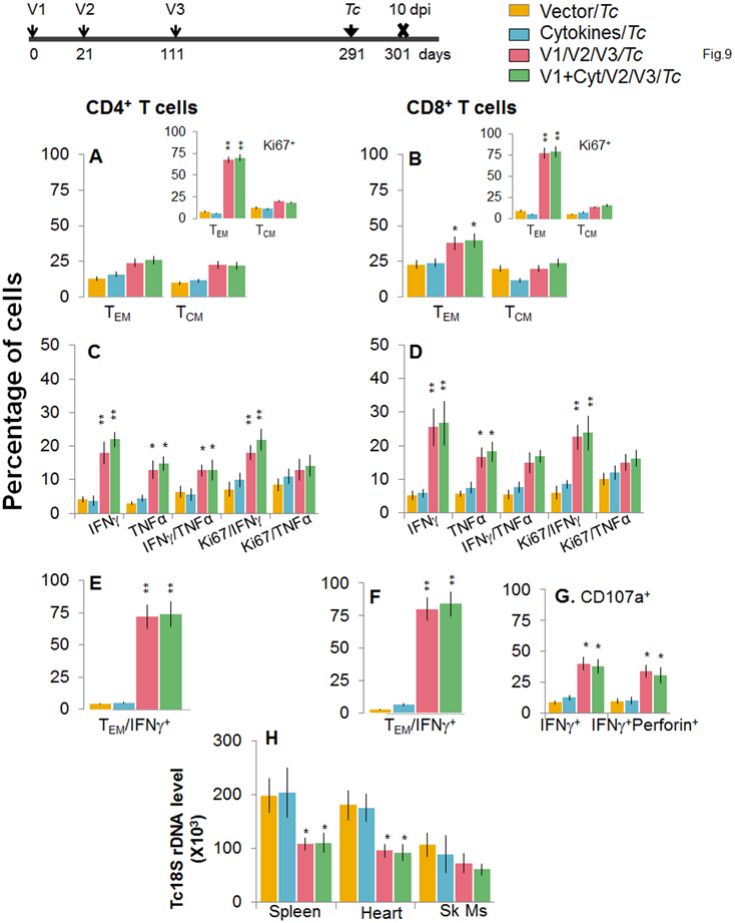
D/P vaccine/bi generated CD8^+^ T_CM_ cells are capable of responding to challenge infection at 180 days post bi. Mice were immunized with D/P vaccine followed by a booster dose as in Fig 4, and then 180 days post bi, infected with *T*. *cruzi*. Mice were harvested 10 days after the challenge infection, and splenocytes were stimulated in the presence or absence of TcG2 and TcG4 recombinant antigens, labeled with fluorescent-conjugated antibodies and analyzed by flow cytometry. **(A&B)** *T*. *cruzi*-specific CD4^+^ ***(A)*** and CD8^+^ ***(B)*** splenic T cell subsets in vaccinated mice infected at 180 days post bi. Insets show *T*. *cruzi*-specific, proliferating (Ki67^+^) T cell subsets in mice challenged at 180 days post bi. **(C-F)** Shown are mean percentage of CD4^+^ ***(C&E)*** and CD8^+^ ***(D&F)*** T cells that responded to *T*. *cruzi* infection by producing IFNγ and/or TNFα cytokines and were proliferative (Ki67^+^, ***C&D***) with a T effector ***(E&F)*** phenotype in mice challenged at 180 days post bi. **(G)** The percentage of *T*. *cruzi*-specific CD8^+^CD107a^+^ that were IFNγ^+^perforin^-^ or IFNγ^+^perforin^+^ in mice that were infected at 180 days post bi. **(H) Tissue parasite burden**. Real time PCR evaluation of *Tc18SrDNA* level, normalized to murine *GAPDH*, in spleen, heart and skeletal muscle tissue of vaccinated/bi mice that were infected at 180 days post bi.

At 180 days post bi, splenic *ex vivo* CD4^+^ and CD8^+^ T_EM_ cells increased by 20–44% only in response to challenge infection (with or without *in vitro* antigenic stimulation) with no change in the T_CM_ population (Fig 9A and 9B). Yet, the effector CD4^+^ and CD8^+^ T cells generated post-challenge infection were poly-functional as was evidenced by 1.8-4-fold increase in IFN*γ*- and/or TNFα- producing CD4^+^T cells (compare Fig 9C with Fig 7C, p<0.01) and up to 2-fold increase in type 1 cytokine-producing CD8^+^T cells of cytolytic phenotype (compare Fig 9D, 9F, and 9G with Fig 7D, 7F, and 7G, p<0.01). The vaccinated mice, challenged at 180 days post bi, were able to achieve a 1.5–1.8-fold control of *T*. *cruzi* infection as compared to that noted in non-vaccinated/infected mice (Figs 9H and S1B, p<0.05). Together, the data presented in Figs 8 and 9 suggested that the D/P vaccine/bi induced T cell immunity was stable, and capable of rapidly expanding with a poly-functional phenotype in response to challenge infection at least until 180 days post bi, and provided significant control of parasite dissemination and replication.

## Discussion

The pathology of Chagas disease presents a complicated and diverse picture in humans [14]. The major complications and destructive evolutionary outcomes of chronic infection by *T*. *cruzi* in humans include ventricular fibrillation, thromboembolism, and congestive heart failure [15,16]. Studies in animal models and human patients have revealed the pathogenic mechanisms found during disease progression, and the features of protective immunity [17]. Parasite-specific CD4^+^T cells are suggested to assist in *T*. *cruzi* control through secretion of Th1 cytokines (e.g., IFNγ, IL2), amplification of the phagocytic activity of macrophages, stimulation of B cell proliferation, and antibody production and differentiation and activation of CD8^+^ T cells (reviewed in [17]). *T*. *cruzi* antigen-specific CD8^+^T cells contribute to parasite control, either by cytolysis of the infected cells or by secretion of Th1 cytokines (IFNγ) that induce trypanocidal activity (reviewed in [18,19]). Accordingly, several antigens (e.g. GP90, cruzipain, GP82, ASP2, TSA1, Tc24) have been tested to elicit protective immunity to *T*. *cruzi* in experimental animals (reviewed in [7,20,21]). A majority of the candidate antigens were selected based upon their potential to be recognized by antibodies in infected mice and have proved to be efficacious as vaccine in providing some degree of protection from *T*. *cruzi* infection. In parallel, efforts to enhance the protective efficacy of subunit vaccines against *T*. *cruzi* have included testing the use of adjuvants, e.g. saponin, CpGODN, IL-12 and GMCSF cytokines; [22] attenuated strain of *Salmonella* [23] or adenovirus [24] for antigen delivery, and heterologous prime-boost protocols [25].

Based upon several studies that we have conducted, we believe TcG2 and TcG4 candidate antigens are an excellent choice for subunit vaccine development, and a heterologous prime/boost approach for vaccine delivery is highly efficacious against *T*. *cruzi* infection. The selected candidates TcG2 and TcG4 tested in this study (and TcG1 tested in other studies) are highly conserved in clinically relevant *T*. *cruzi* strains, expressed (mRNA/protein) in infective trypomastigote and intracellular amastigote stages of *T*. *cruzi*, and released during parasite differentiation in host cell cytoplasm, a characteristic required for antigen presentation for T cell activation [10]. These antigens showed antigen-specific antibody (IgG1, IgG2a and IgG2b) and/or CD8^+^ T cell responses in *T*. *cruzi*-infected mice and dogs. Three candidate antigens (TcG1,TcG2 and TcG4) were also recognized by antibody response in chagasic patients from distinct study sites (Argentina-Bolivia and Mexico-Guatemala) and expressed in diverse strains of the circulating parasites [12,13,26]. Further, we noted that immunization with candidate antigens as a DNA vaccine provided T cell immunity (TcG2 = TcG4>TcG1) that was additive when the antigens were co-delivered and achieved a significant (but modest) control of challenge infection in mice and dogs [10–12]. The delivery of candidate antigens as heterologous prime/boost vaccine [13,27,28] provided protective immunity consisting of parasite- and antigen-specific lytic antibodies and type 1 CD8^+^ cytotoxic T lymphocytes against challenge infection and chronic disease that was significantly better than that observed with DNA-prime/DNA-boost vaccine [10,11]. The enhanced efficacy of a heterologous prime/boost approach for vaccine delivery could be because delivery of antigens as DNA vaccines elicits robust T-cell responses, which are critical for the development of T-cell-dependent antibody responses [29,30], and DNA immunization is also highly effective in priming antigen-specific memory B cells. Delivery of vaccine candidates as recombinant proteins is generally more effective at eliciting antibody responses and may directly stimulate antigen-specific memory B cells to differentiate into antibody-secreting cells, resulting in production of high-titer, antigen-specific antibodies [31,32]. Studies testing the protective efficacy of Tc24, TSA1 (individually or in combination, [33]) or other antigens (reviewed in [7,20,21]) have shown the protection associated with the induction of CD8^+^ T cell activity and IFN-γ production; however, in these studies, challenge infection was conducted immediately after vaccination. To the best of our knowledge, this is the first report documenting that a) a subunit vaccine can be useful in achieving long-term protection against *T*. *cruzi* infection and Chagas disease, and b) the effector T cells can be long-lived and play a role in vaccine elicited protection from parasitic infection.

Approximately, ~30–40% of the infected individuals develop symptomatic clinic disease presented as severe inflammatory myocarditis, and extensive destruction and fibrosis of the heart. *T*. *cruzi* antigen-specific T lymphocytes producing inflammatory cytokines (e.g. IFNγ, IL17) can be detected in most individuals during chronic infection [34]; however, their role in host resistance versus chronic pathology is debated [35–38]. In experimental studies using a variety of genetically modified mice, both innate and adaptive immune responses were found to play an important role in providing resistance to *T*. *cruzi* infection. With regard to innate immune responses, a compromised signaling through toll-like receptors (TLR-3, -7, -9) and their adaptor molecules MyD88 and TRIF, and inhibition of IL-1-activating inflammasomes resulted in increased susceptibility to *T*. *cruzi* infection [39–41]. Likewise, mice genetically deficient in CD4^+^ or CD8^+^ T cell function were extremely susceptible to infection [42], and it was shown that T lymphocytes act by producing type 1 cytokines, such as IFNγ, and CD8^+^ T cell cytotoxicity mediated by perforin is important for resistance against infection [9,25,43,44]. The question then arises as to how to determine that vaccination was effective since both vaccine and *T*. *cruzi* infection elicited potent T cell responses. With respect to this issue, our data in the present study provide clues to the mechanisms relevant to vaccine efficacy. One, immunization with the 2-component D/P vaccine resulted in the generation of antigen-specific, IFNγ-producing CD4^+^ and CD8^+^ T cells and CD8^+^ cytotoxic T lymphocytes that were long-lived and could be further enhanced by bi (Figs 2 and 4). Two, vaccinated mice exhibited an expansion of T cell responses within 7–10 days post-infection. Immune responses elicited by vaccination followed by infection were >3-orders of magnitude higher than that generated by infection only (at 10 dpi) and were mediated by multifunctional lymphocytes abundantly secreting pro-inflammatory cytokines, such as IFNγ and TNFα, capable of translocating the CD107a and perforin molecules to the cell surface, and producing cytotoxic activity against infected target cells. The T cell immunity elicited in D/P vaccinated (± booster-immunized) mice was similar to that noted against self-limiting infections. For example, influenza, LCMV, or *Listeria* have been reported to result in the highest T cell immunity, as measured by the frequency and function of specific CD8^+^ T lymphocytes, within 7–15 days pi [45]. In comparison, in experimental models of *T*. *cruzi* infection and human patients, parasite-specific T cell immunity is delayed (peaks at ≥30 day pi [44,46]); and when improved protective immunity was generated, it correlated with significantly higher numbers of antigen-specific IFN-γ producing total and CD8^+^ T cells that were better able to expand after *in vitro* re-stimulation [47]. Thus, we propose that vaccine-induced protection to *T*. *cruzi* infection and Chagas disease is associated with an increased frequency of highly competent CD8^+^ T lymphocytes at an early stage of parasite infection and replication. Further studies will be required to identify the underlying mechanisms for a delayed T cell response in natural *T*. *cruzi* infection, though a recent study suggests that accelerated apoptosis of CD8^+^T cells, resulting in inability to clear parasites, might be the key reason for chronic disease development [48].

Finally, we propose that our studies challenge the immunological paradigm of vaccine development. It is suggested that following pathogen control (or clearance), 90–95% of the T effector cells reportedly die (contraction phase), leaving behind a population of pathogen-specific CD8^+^ T central memory cells (T_CM_) that undergo antigen-independent/cytokine-dependent homeostatic proliferation which supports their long-term maintenance [45]. Moreover, these cells were found to efficiently increase cytokine and chemokine production, acquire cytotoxic ability and proliferate intensely during recall responses [49]. Thus, after an infectious challenge, heightened precursor frequency, expanded anatomical distribution, enhanced proliferative capacity, and rapid recall ability were reported as the hallmark attributes of protective CD8^+^ T_CM_ cells [50,51]. During chronic *Leishmania* infection, T_EF_ cells were maintained at high frequencies via reactivation of T_CM_ and the T_EF_ themselves and the short lived effector T cells were shown as the critical cells that mediate concomitant immunity [52]. In our studies, we have used a heterologous, prime-boost vaccination regimen consisting of priming with plasmid DNA, followed by a bi with either replication-defective MVA [27,28] or recombinant proteins (this study), in all instances using the TcG2 and TcG4 as vaccine candidates. In all these studies, we observed that our immunization protocol induced a stable pool of functional CD8^+^T_EM_ cells (CD62L^-^CD44^+^), and these cells expanded in response to challenge infection at 14 days pi. In this study, a D/P vaccine (± bi) produced a stable pool of T effector memory (T_EM_) cells that exhibited rapid recall response to challenge infection with a potent increase in antigen-specific inflammatory cytokine production and CTL activity against specific target cells (Figs 3, 5, and 8). We did notice the generation of a strong pool of T_CM_ cells in vaccinated mice after 120 or 180 days post bi, the time period equivalent to chronic infection; however, this pool of T_CM_ was smaller than the T_EM_ cells until 120 days post bi, and became predominant pool only after 180 days post bi (Figs 3, 5, 8, and 9). Others have shown that T_EM_ cells, induced by ASP2 vaccine, were the main source of the anti-parasitic mediators IFNγ and TNFα, and these cells did not need to proliferate (rather increase the function) to provide protective immunity [53].

In patients with severe chagasic disease, a lower frequency of specific CD8^+^T cells with fewer early memory cells (CD45RA^-^, CD27^+^, and CD28^+^) and a higher number of late memory cells (CD45RA^-^, CD27^-^, and CD28^-^) that lack the capability to respond to parasite stimulus was noted [54]. Further, frequency of CD8^+^ T_EM_ lymphocytes remains high for long periods of time after infection, likely due to parasite persistence. Indeed, treatment with an anti-parasite drug in the chronic disease phase led to the contraction of CD8^+^ T cells and a change in their phenotype to CD62L^low^ [55]. Further studies will be required to establish the implications of these observations on the fate of CD8^+^T cells in parasitic infections in the development of new vaccines [56].

In summary, we have demonstrated that our DNA-prime/protein-boost vaccine constituted of TcG2 and TcG4 candidate antigens provides long-lived, rapid recall immunity to *T*. *cruzi* infection. Mice immunized with a 2-component D/P vaccine elicited long-lived CD4^+^ and CD8^+^ effector memory T cells capable of producing type 1 cytokines, and cytotoxic T lymphocyte profile in an antigen-specific manner. The vaccinated mice responded to challenge infection with a rapid and potent expansion of type 1 cytokines producing CD4^+^ and CD8^+^ T cells and cytotoxic T lymphocyte activity against infected target cells, resulting in a >2-3-fold control of acute parasitemia and tissue parasite burden. Importantly, vaccine-induced immunity could be enhanced by bi that helped to maintain a high level of T cell population capable of efficiently expanding and providing an up to five-fold control of an invading pathogen. The vaccine-induced immunity waned slightly after 6 months post bi, but was still sufficient to provide 2-fold control of invading pathogens that, according to mathematical modeling, is sufficient to break the parasite transmission cycle [57] and prevent disease progression [8]. Many of the studies discussed highlight the importance of a preventive or therapeutic vaccine to control *T*. *cruzi* infection by at least decreasing parasite burden, cardiac tissue inflammation and damage or increasing survival if not providing sterile immunity.

## Materials and Methods

### Ethics statement

All animal experiments were conducted following NIH guidelines for housing and care of laboratory animals and in accordance with protocols approved by the Institutional Animal Care and Use Committee (protocol number 08-05-029) at The University of Texas Medical Branch at Galveston.

### *T*. *cruzi* genes and generation of recombinant plasmids, proteins, and viruses

The cDNAs for TcG2 and TcG4 (SylvioX10 isolate, Genbank: AY727915 and AY727917, respectively; >99% homologous to CL Brenner (reference strain) sequences XM_806323 and XM_816508, respectively) [10] were cloned in eukaryotic expression plasmid pCDNA3.1 [10,11]. Plasmids encoding murine IL-12 (pcDNA3.msp35 and pcDNA3.msp40) and GM-CSF (pCMVI.GM-CSF) were previously described [11]. Recombinant plasmids were transformed into *E*. *coli* DH5-alpha-competent cells, grown in L-broth containing 100-μg/ml ampicillin, and purified by anion exchange chromatography by using the Qiagen maxi prep kit (Qiagen, Chatsworth, CA).

The cDNAs for *TcG2* and *TcG4* were cloned in-frame with a C-terminal His-tag into a pET-22b plasmid (Novagen, Gibbstown, NJ). Plasmids were transformed in *BL21* (DE3) pLysS-competent cells, and recombinant proteins purified by using the poly-histidine fusion, peptide-metal chelation chromatography system [13].

The pLW44 vector consists of a green fluorescent protein (GFP) and multiple cloning site (MCS) cassette flanked by a pair of genomic sequences of the Modified Vaccinia Ankara (MVA) virus which allows homologous recombination and incorporation of both GFP and the gene of interest into the deletion III locus of the wild-type MVA genome [58,59]. The cDNAs for *TcG2* and *TcG4* were cloned at the Xma1/Sbf1 sites of pLW44, and recombinant plasmids were transformed and amplified in *E*. *coli*, and purified by using the Qiagen maxi prep kit. BHK-21 cells at 70% confluency (six-well plate) were infected with wild-type MVA (multiplicity of infection: 0.05) for one h, and then transfected with recombinant pLW44 plasmids encoding TcG2 or TcG4 (2 μg DNA) by using Lipofectamine 2000 reagent (Invitrogen, Grand Island, NY). After 48 h of incubation, cells were harvested, and cell lysates added at 10-fold dilutions to new BHK-21 cell monolayers that were then overlaid with 2% methylcellulose (Sigma, St. Louis, MO), and incubated for 48 h. At least three GFP^+^ fluorescent plaques were picked for each recombinant MVA (rMVA). The plaque purification procedure was repeated 4–6 times to ensure removal of wild-type MVA contamination. Purified plaques of rMVA.TcG2 and rMVA.TcG4 were then amplified in BHK-21 cell monolayers, and viral pellets were stored in 1 mM Tris-HCl (pH 9) at -80°C [27].

### Immunization and challenge infection in mice

*T*. *cruzi* trypomastigotes (Sylvio X10/4 strain) were maintained and propagated by continuous *in vitro* passage in C2C12 cells. C57BL/6 female mice (6-week-old) were obtained from Harlan Labs (Indianapolis, IN). Mice (7-8-weeks-old) were immunized with the DNA prime dose consisting of the TcG2- and TcG4-encoding plasmids with or without IL-12- and GM-CSF-expression plasmids (25 μg each plasmid DNA/mouse, intramuscularly). Twenty-one days later, mice were given recombinant proteins (TcG2 and TcG4, 25 μg of each protein emulsified in 5 μg saponin/100 μl PBS/mouse, intra-dermally). Mice were harvested at 14 and 120 days after vaccination to determine early and long-term vaccine-induced T cell immunity, respectively. In some experiments, 90 days after the DNA-prime/protein-boost (D/P) vaccination, mice were given a booster immunization (bi) with recombinant proteins as above, and then sacrificed at 14, 120 and 180 days later to evaluate if bi provided stable, long-term anti-*T*. *cruzi* T cell immunity.

To evaluate the recall response to *T*. *cruzi*, mice in each group, i.e., 120 days after D/P vaccination or 14, 120, and 180 days after D/P/P vaccination, were challenged with *T*. *cruzi* (10,000 trypomastigotes/mouse, i.p.), and sacrificed 10 days post infection (dpi). Some mice were also sacrificed at >90 dpi to evaluate the vaccine efficacy in controlling chronic parasite burden.

### *In vitro* stimulation of splenocytes, T cell characterization, and intracellular cytokines

Single-cell suspensions of spleen cells were prepared and cell number counted (S1 Table). Splenocytes (10^5^ cells/100 μl RPMI) were distributed in 24-well plates, and incubated in the presence of Con A (5 μg/ml), recombinant proteins (10 μg/ml), or *T*. *cruzi* trypomastigotes lysate (TcTL, 25 μg protein/ml) at 37°C, 5% CO_2_ for 48 h. Un-stimulated or *in vitro-* stimulated splenocytes were washed in staining buffer (2% BSA/0.02% sodium azide in PBS) and incubated for 15 min in Fc Block (anti-CD16/CD32; BD Pharmingen). To evaluate the surface staining of effector and memory markers, we incubated the cells with the fluorescence- conjugated PE-αCD4, FITC-αCD8, PerCPCy5.5-αCD62L and APC-αCD44 antibodies (BD Pharmingen) for 30 min at 4°C in the dark. Then cells were washed twice in PBS and fixed in 2% paraformaldehyde. Fluorescent cells were visualized by using a FACSCalibur Cell Analyzer (BD Biosciences), acquiring >30,000 events in a live lymphocyte gate, and further analyzed by using FlowJo software (version 7.6.5, Tree-Star, San Carlo, CA) [27].

For the measurement of intracellular cytokines, splenocytes were stimulated as above except that brefeldin A (10-μg/ml, Sigma) or monensin (5 μg/ml) (BD Pharmingen) was added for the final 6 h of culture to block protein secretion. Cells were labeled with PE-αCD4 and FITC-αCD8 antibodies, fixed with 2% paraformaldehyde, re-suspended in 100 μl permeabilization buffer (0.1% saponin/1% FBS in PBS), and then utilized for intracellular staining with APC-αIL4, PerCPCy5.5-αIL10, e-Fluor-αIFNγ, Cy5-αTNFα and PerCP-Cy5.5-αKi67 antibodies (0.5-2-μg/100-μl, e-Biosciences). In some experiments, splenocytes were also incubated with APC-anti-perforin or Alexa-Fluor 488-anti-CD107 antibodies to determine the cytolytic activity of the activated/proliferating T cell subpopulations. Samples were analyzed by flow cytometry as above. In all experiments, cells stained with isotype-matched IgGs were used as controls [13,27].

### Cytotoxic T lymphocyte (CTL) activity

Splenocytes (5×10^6^ cells/2 ml/well in 24-well plates) were incubated with recombinant TcG2 or TcG4 proteins (10-μg/ml) at 37°C, 5% CO_2_ for 4–5 days to generate effector T cells_._ Semi-confluent EL-4 monolayers were exposed to rMVA encoding TcG2 or TcG4 (10 pfu/cell) for 1 h (controls: WT MVA), washed, and incubated in complete medium for 12 h at 37°C. Target EL-4 cells infected with GFP^+^ rMVA were co-cultured with effector cells (Effector: target cell ratio: 80:1–20:1) in 200 μl of RPMI medium at 37°C, 5% CO_2_ for 4 h. Cells were mixed with 2.5 mM EDTA to reduce the number of cell-cell conjugates, and 5 μl propidium iodide (PI) to discriminate viable and nonviable cells, and analyzed by flow cytometry for a fixed period of 60 sec/sample. The forward scatter (FSC) acquisition threshold was set to include nonviable events. The CTL activity was calculated using [GFP^+^ target cells after incubation with effector cells x 100 / Total GFP^+^ target cells].

### Blood and tissue parasite burden

Blood DNA was isolated with a QiAamp Blood DNA mini kit (Qiagen, Chatsworth, CA). Skeletal muscle, spleen, and heart tissue (50 mg) were subjected to proteinase K lysis, and total DNA was purified by phenol/chloroform extraction and ethanol precipitation. Total DNA (50 ng) was used as a template, and real-time PCR performed on an iCycler thermal cycler with SYBR Green Supermix (Bio-Rad) and *Tc18SrDNA*-specific oligonucleotides. Data were normalized to murine *GAPDH* and fold change in parasite burden (i.e. *Tc18SrDNA* level) calculated as 2^-ΔCt^, where ΔC_t_ represents the C_t_ (infected)—C_t_ (control) [60,61].

### Statistical analysis

Data are expressed as mean ± SD (n = 8/group, triplicate observations per experiment). Data were analyzed by the Student’s *t* test (comparison of 2 groups) and 1-way analysis of variance (ANOVA) with Tukey’s post-hoc test (comparison of multiple groups) by using an SPSS (version 14.0, SPSS Inc, Chicago, Illinois) or Graph Pad InStat ver.3 software. Significance is presented as *p<0.05, **p<0.01, or ***p<0.001 (vaccinated versus non-vaccinated or vaccinated/infected versus non-vaccinated/infected).

## Supporting Information

S1 Table Splenic cell count. C57BL/6 mice were immunized with empty vector, cytokines only, or D/P vaccine (V1 dose: TcG2- and TcG4-encoding plasmids ± IL-12- and GMCSF-expression plasmids; and V2 dose: recombinant TcG2 and TcG4 proteins). Mice were harvested at day 14 post-vaccination (pv, as in Fig 1), 120 pv (as in Fig 2) or infected at 120 days pv and harvested 14 days later (as in Fig 3). In some cases, after the D/P vaccination, mice were given a booster immunization (bi) of recombinant TcG2/TcG4 proteins. Mice were harvested at 14 day booster immunization (dbi, as in Fig 4) or infected at 14 dbi and harvested 10 days later (as in Fig 5). To examine the longevity of vaccine-primed immunity, mice were also harvested at 120 dbi (as in Fig 6) or 180 dbi (as in Fig 7). Booster-immunized mice were infected with *T*. *cruzi* at 120 dbi (as in Fig 8) or at 180 dbi (as in Fig 9) and harvested 10 days later. Single cell suspension of whole spleen was made and cell number counted by light microscopy (n = 5 per group per experiment).
(DOCX)

S1 Fig Blood parasite burden. **(A)** C57BL/6 mice were immunized with an empty vector, cytokines only, or two dose vaccine, and, infected with *T*. *cruzi*, as in Fig 3 (total 151 days). **(B)** Mice were immunized with V1, V2, and V3 doses of vaccine and infected with *T*. *cruzi* as in Fig 5 (total 135 days), Fig 8 (total 241 days) and Fig 9 (total 301 days). In all experiments, mice were harvested 10 days post-infection. Total DNA was isolated from blood of vaccinated/infected mice and real time PCR amplification of *Tc18SrDNA* sequence was performed. Bar graphs show the *Tc18SrDNA* level normalized to murine *GAPDH*.
(TIF)
