# Supplementary material for: A Two-Component DNA-Prime/Protein-Boost Vaccination Strategy for Eliciting Long-Term, Protective T Cell Immunity against Trypanosoma cruzi
Source: PLoS Pathog. 2015 May 7;11(5):e1004828. doi: 10.1371/journal.ppat.1004828 (PMC4423834; doi:10.1371/journal.ppat.1004828)
Supplement: S1 Table — C57BL/6 mice were immunized with empty vector, cytokines only, or D/P vaccine (V1 dose: TcG2- and TcG4-encoding plasmids ± IL-12- and GMCSF-expression plasmids; and V2 dose: recombinant TcG2 and TcG4 proteins). Mice were harvested at day 14 post-vaccination (pv, as in Fig 1), 120 pv (as in Fig 2) or infected at 120 days pv and harvested 14 days later (as in Fig 3). In some cases, after the D/P vaccination, mice were given a booster immunization (bi) of recombinant TcG2/TcG4 proteins. Mice were harvested at 14 day booster immunization (dbi, as in Fig 4) or infected at 14 dbi and harvested 10 days later (as in Fig 5). To examine the longevity of vaccine-primed immunity, mice were also harvested at 120 dbi (as in Fig 6) or 180 dbi (as in Fig 7). Booster-immunized mice were infected with T. cruzi at 120 dbi (as in Fig 8) or at 180 dbi (as in Fig 9) and harvested 10 days later. Single cell suspension of whole spleen was made and cell number counted by light microscopy (n = 5 per group per experiment). (DOCX) [file ppat.1004828.s001.docx]

**Table S1. Splenic cell count.**

|  | **Time-line (dpv/dbi/dpi)** | **Splenic cell count x 10^6^** | | | |
| --- | --- | --- | --- | --- | --- |
|  |  | **Vector only** | **Cytokine only** | **V1/V2** | **V1+Cyt/V2** |
| **Fig.1** | **14 dpv** | 70±8 | 80±12 | 120±18*** | 134±21*** |
| **Fig.2** | **120 dpv** | 65±10 | 72±8 | 138±36*** | 144±45*** |
| **Fig.3** | **120 dpv/10 dpi** | 1220±102 | 1542±116 | 1850±142*** | 2032±135*** |
|  |  | **Vector only** | **Cytokine only** | **V1/V2/V3** | **V1+Cyt/V2/V3** |
| **Fig.4** | **14dbi** | 62±12 | 76±8 | 180±54*** | 160±40*** |
| **Fig.5** | **14dbi/*Tc*** | 1324±152 | 1620±135 | 2135±168*** | 2200±194*** |
| **Fig.6** | **120 dbi** | 60±12 | 72±8 | 202±20*** | 195±24*** |
| **Fig.7** | **180 dbi** | 58±11 | 64±11 | 215±21*** | 225±32*** |
| **Fig.8** | **120 dbi/*Tc*** | 1435±145 | 1590±126 | 2016±182*** | 2045±155*** |
| **Fig.9** | **180 dbi/*Tc*** | 1205±158 | 1612±188* | 2024±201*** | 2000±186*** |

C57BL/6 mice were immunized with an empty vector, cytokines only, or D/P vaccine (V1 dose: TcG2- and TcG4-encoding plasmids ± IL-12- and GMCSF-expression plasmids; and V2 dose: recombinant TcG2 and TcG4 proteins). Mice were harvested at day 14 post-vaccination (pv, as in Fig.1), 120 pv (as in Fig.2) or infected at 120 days pv and harvested 14 days later (as in Fig.3). In some cases, after the D/P vaccination, mice were given a booster immunization (bi) of recombinant TcG2/TcG4 proteins. Mice were harvested at 14 day booster immunization (dbi, as in Fig.4) or infected at 14 dbi and harvested 10 days later (as in Fig.5). To examine the longevity of vaccine-primed immunity, mice were also harvested at 120 dbi (as in Fig.6) or 180 dbi (as in Fig.7). Booster-immunized mice were infected with *T. cruzi* at 120 dbi (as in Fig.8) or at 180 dbi (as in Fig.9) and harvested 10 days later. Single cell suspension of whole spleen was made and cell number counted by light microscopy (n=5 per group per experiment).
