# Supplementary figures and images for: A Two-Component DNA-Prime/Protein-Boost Vaccination Strategy for Eliciting Long-Term, Protective T Cell Immunity against Trypanosoma cruzi
Source: PLoS Pathog. 2015 May 7;11(5):e1004828. doi: 10.1371/journal.ppat.1004828 (PMC4423834; doi:10.1371/journal.ppat.1004828)

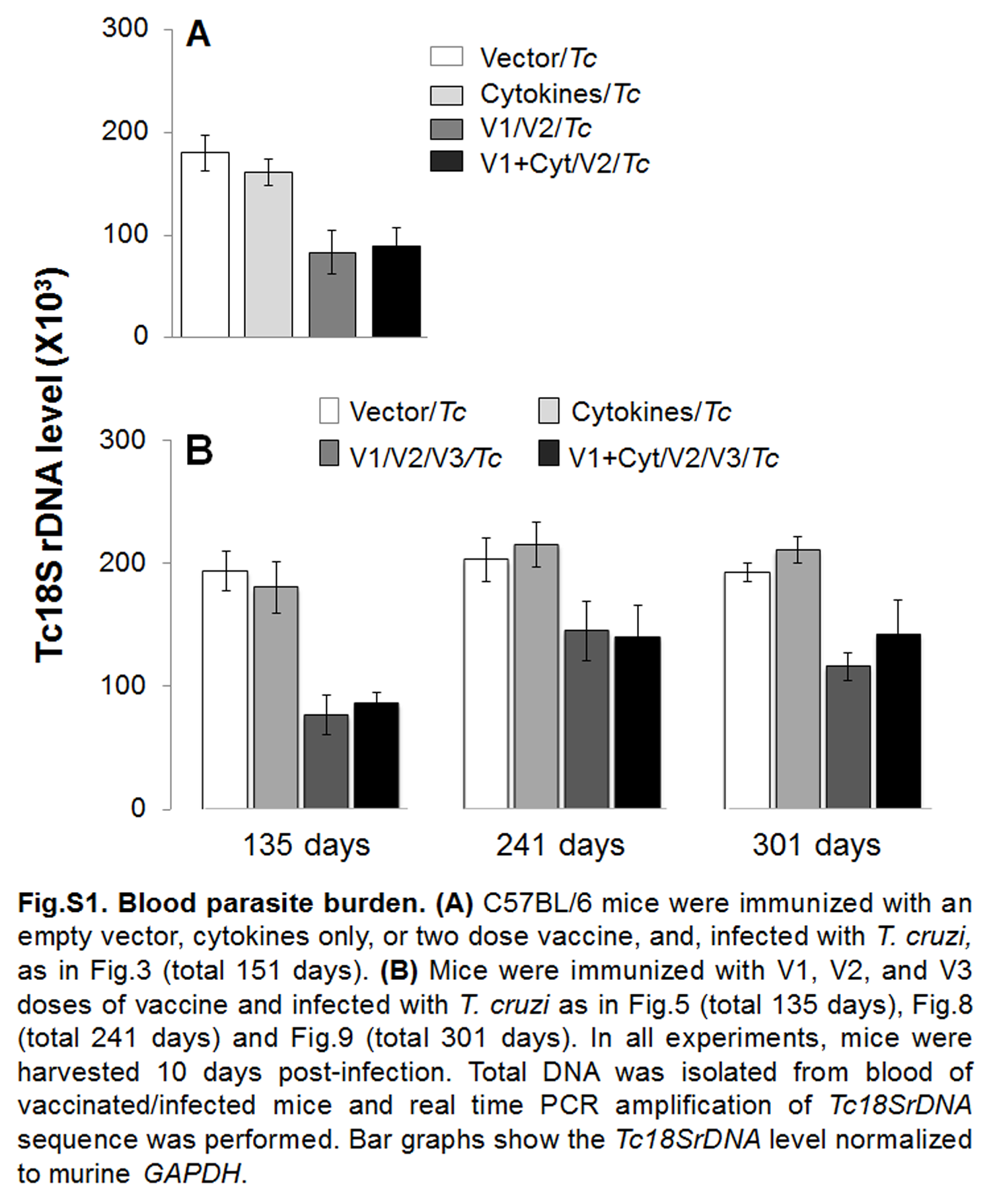

Supplement: S1 Fig — (A) C57BL/6 mice were immunized with an empty vector, cytokines only, or two dose vaccine, and, infected with T. cruzi, as in Fig 3 (total 151 days). (B) Mice were immunized with V1, V2, and V3 doses of vaccine and infected with T. cruzi as in Fig 5 (total 135 days), Fig 8 (total 241 days) and Fig 9 (total 301 days). In all experiments, mice were harvested 10 days post-infection. Total DNA was isolated from blood of vaccinated/infected mice and real time PCR amplification of Tc18SrDNA sequence was performed. Bar graphs show the Tc18SrDNA level normalized to murine GAPDH. (TIF) [file ppat.1004828.s002.tif]
